# Consistent Temperature Coupling with Thermal Fluctuations of Smooth Particle Hydrodynamics and Molecular Dynamics

**DOI:** 10.1371/journal.pone.0051989

**Published:** 2012-12-26

**Authors:** Georg C. Ganzenmüller, Stefan Hiermaier, Martin O. Steinhauser

**Affiliations:** Fraunhofer Institute for High-Speed Dynamics, Ernst-Mach-Institut, Freiburg, Germany; University of Illinois, United States of America

## Abstract

We propose a thermodynamically consistent and energy-conserving temperature coupling scheme between the atomistic and the continuum domain. The coupling scheme links the two domains using the DPDE (Dissipative Particle Dynamics at constant Energy) thermostat and is designed to handle strong temperature gradients across the atomistic/continuum domain interface. The fundamentally different definitions of temperature in the continuum and atomistic domain – internal energy and heat capacity versus particle velocity – are accounted for in a straightforward and conceptually intuitive way by the DPDE thermostat. We verify the here-proposed scheme using a fluid, which is simultaneously represented as a continuum using Smooth Particle Hydrodynamics, and as an atomistically resolved liquid using Molecular Dynamics. In the case of equilibrium contact between both domains, we show that the correct microscopic equilibrium properties of the atomistic fluid are obtained. As an example of a strong non-equilibrium situation, we consider the propagation of a steady shock-wave from the continuum domain into the atomistic domain, and show that the coupling scheme conserves both energy and shock-wave dynamics. To demonstrate the applicability of our scheme to real systems, we consider shock loading of a phospholipid bilayer immersed in water in a multi-scale simulation, an interesting topic of biological relevance.

## Introduction

In this letter, we report a thermodynamically consistent coupling scheme between the atomistic simulation method Molecular Dynamics (MD), and the macroscopic continuum simulation technique Smooth Particle Hydrodynamics (SPH). The coupling of such methods, which operate on vastly different time- and length-scales, is referred to as *scale-bridging* or *multi-scale simulation* [1], [2]. This very active topic of current research allows physicists and material scientists to gain insight into phenomena, where microscopic processes determine macroscopic effects. One of the most important area of application includes crack nucleation and propagation, leading to material fatigue and, eventually, failure [3]–[5].

Over the past two decades, numerous different continuum-atomistic coupling strategies have been devised [6]–[15], all of which offer advantages in specific situations, but lack the necessary generality to be applied as a standard tool. In particular, the problem of describing heat exchange across the continuum-atomistic interface has not yet been solved to a satisfying degree. The existing body of literature considering coupling of atomistic and continuum scales has almost exclusively considered isothermal processes, for which heat exchange can be neglected. However, a huge range of interesting problems are of transient, discontinuous and therefore non-equilibrium nature: shock induced deformation, or unsteady shear flow are examples. These processes involve, by definition, steep gradients of temperature and density which need to be accurately described by a scale-bridging simulation method in order to allow for faithful modeling.

The conceptual problem with formulating heat flux between continuum and atomistic domains is rooted in the fundamentally different representation of these domains: In the continuum approach, one discretizes a continuous field of state variables at discrete spatial locations (integration nodes), between which the fluxes of heat and mass are numerically evaluated according to, e.g. the set of Navier-Stokes equations. They are closed with a consititutive equation of state which links density and pressure, along with a prescription for transport properties such as viscosity. The atomistic approach taken in MD is to use particles which carry mass and interact with each other via distance- and orientation-dependent forces. In MD, momentum exchange is effected through the many-body interactions between particles, which determines the time evolution of position and velocity. Because no other degrees of freedom except those associated with the particle kinetic energy are present, temperature depends solely on momentum, as indicated by the equipartition theorem:
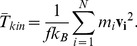
(1)


Here, 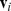 and 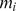 are velocity and mass of particle 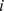, while 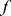 and 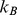 are the system’s number of degrees of freedom (we consider only translation here), and Boltzmann’s constant, respectively. The number 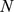 denotes the ensemble size, which has to be taken large enough in order to be meaningful in a macroscopic interpretation. We have explicitly marked this temperature expression with the index “
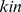
” to emphasize that the appropriate temperature definition in MD is a *kinetic temperature*, and with an overbar, to remind of its average nature. In contrast, the continuum expression for temperature is based on the concept of internal energy 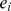 and heat capacity 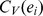, both defined individually for the corresponding integration node 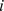:
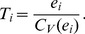
(2)


Thus, temperature in an atomistic description relates to average particle momentum, while it is just a state variable in the continuum representation, with no relation to the momentum of the corresponding continuum integration node.

The challenge for a scale-bridging simulation technique is to effect both momentum exchange and heat flux across the continuum-atomistic interface. Momentum exchange can be incorporated relatively easily by choosing a spatial node discretization density in accordance with the number density of the MD particles, and using MD forces between continuum nodes and MD particles. However, in an adiabatic situation where the MD particle’s velocities are not coupled to an external thermostat, this approach leads to reduction of the temperature in the MD domain, and eventually freezing, at the cost of an increased temperature in the continuum domain. This behavior is due to the momentum exchange, which accelerates the integration nodes. These nodes are subsequently slowed down by the inherent inner friction (viscosity) operating in the continuum domain, unidirectionally converting the nodes’ kinetic energy into internal energy. What is lacking on the continuum side are the thermal fluctuations, which convert back and forth between internal energy and node velocities in accordance with the appropriate Boltzmann distribution. Various approaches have been published to incorporate these fluctuations into a continuum description, with the work of Español [16] being the currently most stringent and promising, although these concepts appear not to have been used yet for adiabatic multi-scale simulations.

Here, we present a new and conceptually straightforward coupling algorithm which combines macroscopic heat diffusion between continuum and atomistic domains with a local thermostat working in the MD domain. This thermostat effects the appropriate conversion between heat and MD particle velocity, generating thermal fluctuations in accordance with a Boltzmann-weighted fluctuation-dissipation theorem.

The validity of our new coupling scheme is demonstrated by showing that essential observables, such as the Maxwell-Boltzmann distribution of momenta, and correct propagation of a shock wave across continuum and MD domains, free of numerical artifacts, are fulfilled. To demonstrate the actual usefulness of the coupling scheme, we consider a multi-scale simulation of a biological system: Shock-loading of a phospholipid bilayer immersed in water.

### Description of the Algorithm

A correct continuum-MD coupling algorithm needs to describe heat flux between the two domains such that the continuum variable internal energy 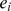 is locally linked to MD particle velocity. Such a thermostat is given by the formulation of Dissipative Particle Dynamics at constant Energy (DPDE) due to Avalos and Mackie [17], and Español [18], which is applied to the MD particles. DPDE is an extension to Dissipative Particle Dynamics (DPD) [19], which is a coarse-graining (CG) technique for MD. DPD mimics the complex particle dynamics of a fully detailed system by incorporating stochastic fluctuations in the equations of motion of the corresponding CG system which employs comparatively less particles. The amplitude of these fluctuations is controlled by a fluctuation-dissipation theorem which depends on the temperature that is taken as a system-wide constant. Thus, DPD is strictly an *isothermal* method. In contrast, DPDE associates a *local internal temperature* 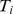 with each particle 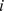, given by an internal energy variable 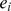 and a heat capacity 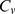, such that 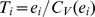. DPDE is therefore a *mesoscopic* simulation method, incorporating both a continuum description of temperature (c.f. Eq. 2) and per-particle degrees of freedom like MD. With only a *local* temperature dependence, DPDE is able to describe temperature gradients as needed for the simulation of non-equilibrium phenomena, such as shock-wave propagation in a medium. The here introduced coupling algorithm works by prescribing macroscopic heat flow between the continuum domain’s internal energy and the DPDE particles’ internal energies. DPDE then establishes an equilibrium between the MD particle velocity 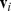 and the local internal temperature, such that the time averages of 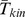 and 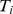 are equal. In conjunction with momentum exchange realized by conventional MD pair forces acting between continuum domain integration nodes and DPDE particles, both heat and mechanical energy fluxes are described.

**Figure 1:**
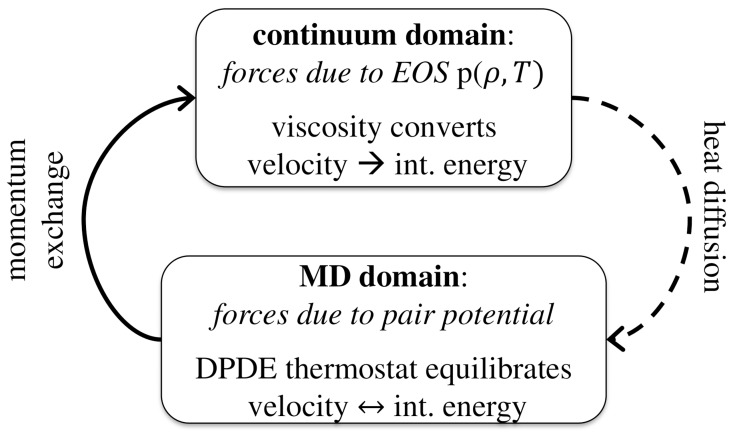
Sketch of the proposed continuum-MD coupling algorithm. Note that – at equilibrium – the flow is directed as shown because continuum viscosity irreversibly transforms nodal velocity into internal energy (see text for a detailed discussion).

We thus propose a coupling strategy along the following lines: In the continuum domain, the time evolution of the system is dictated by constitutive equations, and the domain is spatially discretized into points, which serve as integrations nodes for the partial differential equations of continuum mechanics. To this domain, a region with atomistic length-scales and corresponding particle dynamics is coupled. This region is described by classical MD, i.e., interactions between pairs of particles following microscopic laws are used to describe physical properties. Because the concept of temperature is fundamentally different in both domains – average particle velocity in MD vs. internal energy in the continuum – the local DPDE thermostat is used in the MD domain to achieve dynamic equilibrium between both temperature definitions. In addition, macroscopic heat conduction is used to model heat diffusion between MD/DPDE particles and continuum integration nodes. Figure 1 gives a schematic overview of our proposed continuum-MD coupling scheme. principle. In the following, we will introduce the required expressions for the pair forces, heat flow, and continuum discretization.

#### The DPDE local thermostat

The DPDE pair interaction, which acts between pairs of MD particles, is given by the thermal force
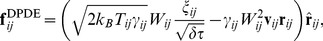
(3)with velocities difference 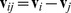 and distance 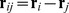. 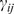 is a scaling factor which controls the strength of the interaction, 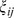 a random variable of Gaussian distribution, 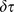 the size of the time-step of the integration scheme, and 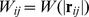 a weighting kernel describing the variation of the interaction as a function of distance. 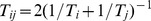 is the average local temperature of the interacting particles. The effect of the thermal force is accelerating or slowing down pairs of particles such that the particle velocity is in agreement with the local temperature, c.f. Eq. (1) and Eq. (2). The time-evolution of the particles’ internal energy follows from imposing conservation of total energy, but needs to be evaluated using Itô calculus [18] due to the stochastic nature of the force Eq. (3),



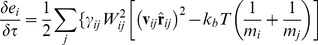


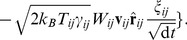
(4)


#### Discretization of the continuum

In the following, we restrict ourselves to SPH for describing the continuum domain, although the here proposed coupling algorithm is also suitable to other continuum discretization methods. Coupling of SPH and MD is most appealing from a conceptual perspective, as both are mesh-free methods, with their time-evolution governed by Newton’s equations of motion. In SPH, a continuum domain is discretized by employing a number of Lagrangian integration nodes carrying mass 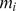 and internal energy 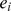. Together with a prescription for the heat capacity 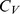, a local temperature 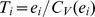 is defined akin to the DPDE case. The mass density at each integration node (termed SPH particle henceforth) is calculated from
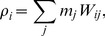
(5)where the sum extends over all integration nodes within the range of the weighting function 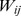, centered at integration node 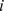. With temperature and mass density defined, a yet to be defined equation of state provides the pressure 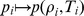 from which the forces between integration nodes follow [20]:

In the above equation, 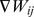 is the spatial derivative of the weighting function 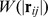 with respect to 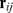. 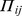 is a viscous term that is based on the linear part of the standard artificial viscosity due to Gingold and Monaghan [21], cast into a form which depends on the effective kinematic viscosity 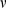 [22]:
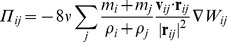
(7)


The corresponding change in internal energy of particle 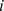 per time-step due to the SPH forces is [20]:
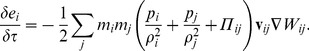
(8)


#### Macroscopic heat flow

The continuum expression for heat conduction is
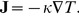
(9)


The flux of internal energy (heat) 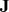 is proportional to the temperature gradient 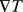, with conduction coefficient 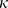. The solution for the time evolution of this flow is a diffusion equation, which has been cast into a SPH discretized form by Cleary [23]:
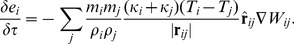
(10)


The conduction coefficient 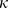 is related to the heat diffusion coefficient 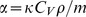.

## Results

### Verification Examples

In order verify the approach proposed here, we consider a Lennard-Jones (LJ) fluid defined by the familiar pair interaction
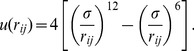
(11)


Due to the computational efficiency of this pair potential, its EOS has been determined with great accuracy. We therefore have the fortunate situation that both continuum and MD methods yield the same results, which renders this an ideal test case for studying the coupling of these methods. On the continuum side, we use a LJ EOS due to Ree [24], discretized using SPH. For the MD/DPDE particles, interactions are given by the pair potential Eq. (11) and DPDE forces given in Eq. (3). We employ the standard system of reduced units [25], defined by the unit of energy  and the unit of length 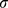. As example of these units we note the reduced temperature 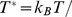, reduced number density 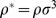, reduced pressure 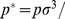, and reduced simulation time 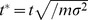. The LJ pair potential is cut and shifted at 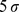 and the DPDE amplitude is set to 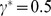. For the weighting function we employ Lucy’s choice [26]

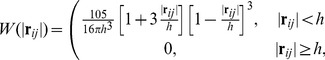
(12)with 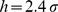. Heat conduction according to Eq. (10) exists across all particles, with a thermal diffusivity coefficient 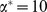. The effective kinematic viscosity acting between SPH particles was set to 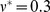, in agreement with the transport properties measured by MD simulations of the LJ potential at the state points 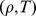 that are considered here. We note that simulation results are rather insensitive to the specific choice of 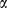 and 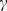, provided that the DPDE conversion rate of internal/kinetic energy is faster than the dissipation rate due to the viscosity, such as not to create a bottleneck in the flow depicted in Fig. 1. The cross-interaction between SPH and MD/DPDE particles is simply a weighted sum of both MD/DPDE and SPH forces, with weighting coefficients of one half for each type of interaction. All particles are assigned a value for the reduced mass of 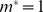. Integration of the equations of motion is performed using the Velocity-Verlet algorithm, implemented in the open source MD code LAMMPS [27], which makes this continuum coupling algorithm generally available at time of publication.

#### Equilibrium properties

**Figure 2:**
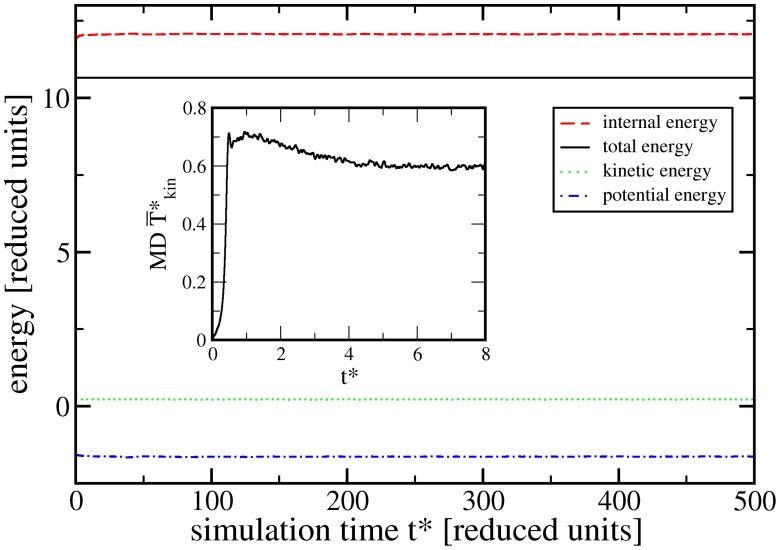
Time-evolution of potential, kinetic, and internal energies corresponding to the temperature equilibrium problem. The inset shows the initial equilibration of the MD/DPDE kinetic temperature.

With the above defined force properties, a system with 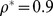, consisting of 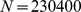 particles, was set up on a simple cubic lattice with 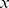, 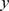, 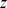 extents of 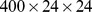 lattice points, centered about the coordinate (0,0,0). From these, the central 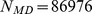 lattice points were assigned to be of MD/DPDE type, and the rest of SPH type. Periodic boundary conditions were used along all three Cartesian directions. All particles were assigned a constant heat capacity 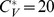 and internal energy 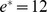, yielding an initial internal temperature 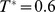 and zero initial velocity. With these parameters, the value of the thermal conductivity becomes 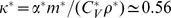. Using a time-step 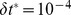, the system was then run for 5 million time steps. This choice of time-step is required for good energy conservation within the MD/DPDE region; the corresponding Courant-Friedrichs-Lewy criterion for the SPH region predicts 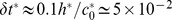, given that the equilibrium speed of sound at the considered state point is 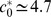. After a time 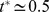, the velocity average within the MD/DPDE particle region first attains a value corresponding to 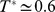, followed by a mild overshoot which is damped out after 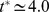. Fig. 2 shows the time-evolution of kinetic, potential, and internal energies as well as the kinetic temperature of the MD/DPDE particles only. We note that conservation of total energy in the DPDE algorithm is not as good as one usually expects from MD and Velocity-Verlet time integration [25] because Eq. (4) is only accurate to 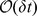. Nevertheless, this very long run conserves total energy with an accuracy of 100 parts per million, an error which is hardly discernible from Fig. 2.

Following equilibration, data were accumulated in a histogram of particle velocities (bin width 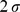), resolved along the 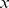-coordinate. As discussed in the introduction, we expect these velocities to correspond directly to the temperature definition within the MD region, according to Eq. (1). Neglecting any change in potential energy (as justified by Fig. 2), this temperature can be estimated a priori by redistributing the internal energy among the internal heat capacity and the translational degrees of freedom of the MD particles:
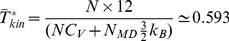
(13)


**Figure 3:**
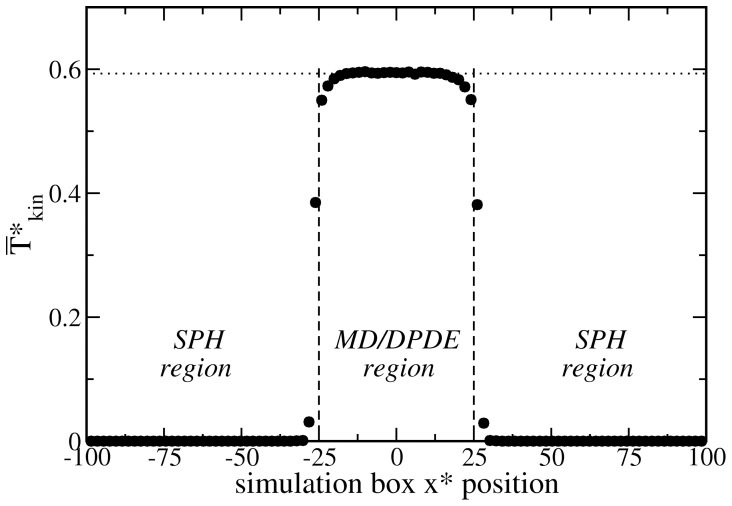
Profile of the kinetic temperature 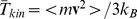 along the simulation box 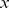-axis. The dotted line at 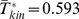 shows the estimate for the MD particles from Eq. (13); full symbols are simulation averages over bin widths of 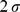.

In contrast, we expect zero particle velocity within the continuum domain, due to the irreversibility of macroscopic viscosity. Figure 3 shows that these expectations are met with a steep change of the particle velocities at the interface between MD and SPH regions, smoothed over a few particle spacings. Within the center of the MD region, a kinetic temperature corresponding very closely to the estimate given above is reached.

**Figure 4:**
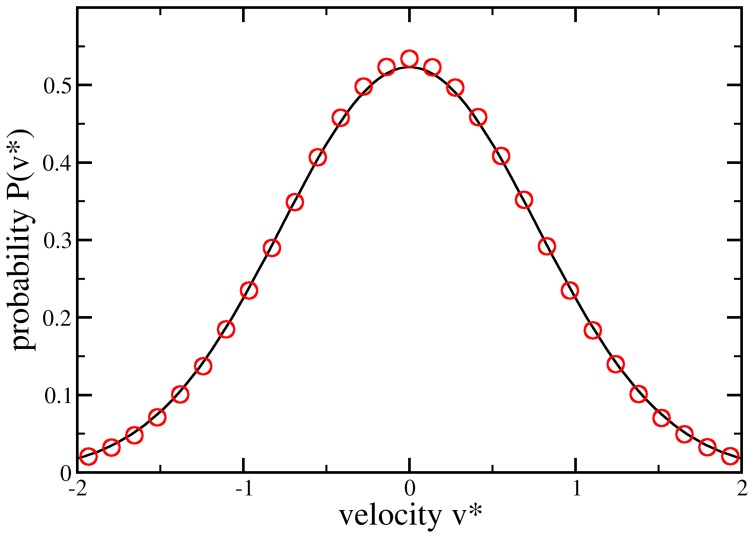
Comparison of the Maxwell-Boltzmann velocity distribution, Eq. 14, (full line) with the measured results for the MD particles (symbols).

We next consider the probability distribution of the MD/DPDE particles’ velocities. From equilibrium thermodynamics, we expect this distribution to be given by the Maxwell-Boltzmann distribution
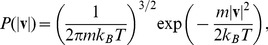
(14)a necessary condition for correct microscopic dynamics. As shown in Fig. 4, the simulation results accurately follow the theoretical prediction.

#### Dynamic test case

**Figure 5:**
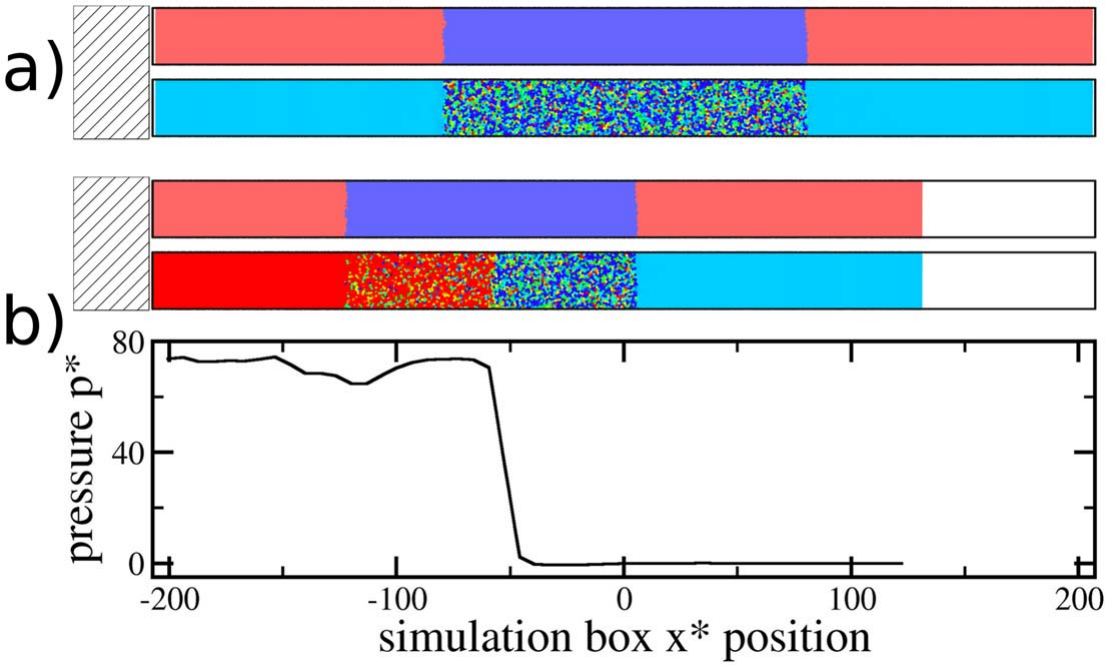
Snapshots of shock-wave propagation. Part a) shows the 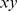-projection of the simulation box; the hatched area on the left denotes a hard wall, i.e., the momentum-reflecting mirror (see text). All particles move left with particle velocity of 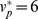. The snapshot is shown in two different representations, with the upper part indicating particle type (red = SPH, blue = MD), and the lower part representing a color-coding of local pressure (blue = low pressure, red = high pressure). Note that the average pressure in the MD region equals the pressure in the SPH region. Part b) shows the simulation at 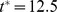, after the particles have moved and compressed against the left wall, with a corresponding pressure increase. The shock-wave front has reached the center of the MD particle region, as indicated by the pressure profile.

**Figure 6:**
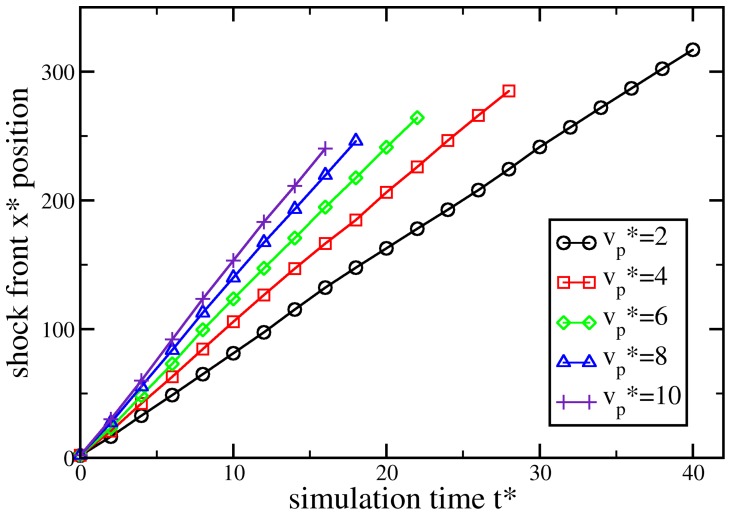
Propagation of shock-waves through a system simulated using both SPH and MD/DPDE. The shock-waves travel at of different speeds, corresponding to different particle velocities 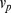, and therefore reach the end of the simulation box at different times. Shock wave speeds range from 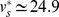 to 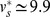, while the speed of sound in the corresponding equilibrium system is 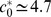.

In order to demonstrate the applicability of our proposed coupling scheme to situations far away from equilibrium, we consider generation and propagation of a shock-wave through a system, which is partially described by continuum SPH and partially by MD. This case provides a test for the applicability of the algorithm to steep gradients in pressure, density, and temperature. The initial system configuration is as described above, with the only difference that momentum-reflecting mirrors are applied at the 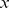-axis boundaries, and that MD/DPDE particles are given initial random velocities corresponding to 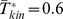. Following an equilibration run of 100000 time-steps, a shock-wave is initiated by adding a constant velocity -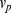 to the 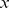-component of each particle’s velocity. Thus, the entire system is treated as a bar which impacts a rigid wall. This induces a shock-wave as particles hit and pile up against the left wall (see Fig. 5). The shock-wave front, i.e., the discontinuity at which the mass density increases suddenly, moves with speed 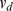 to the right, such that the shock-front velocity is given in a co-moving frame as 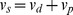. The shock-wave first propagates through the SPH region, then through the MD/DPDE region, and again through an SPH region. Figure 6 shows the shock front location for different particle velocities. The gradient of these data corresponds to the shock-wave velocity, which is almost exactly conserved for all particle velocities considered. This result indicates that our coupling algorithm is stable under simulation conditions which involve steep temperature and pressure gradients.

### Application to Shock Induced Damage in a Phospholipid Bilayer

In this section we demonstrate the applicability of our coupling scheme to real systems. We investigate the interaction of strong shock-waves with a model system of an eucaryotic cell membrane. To this end, we consider a phospholipid bilayer, composed of Dipalmitoylphosphatidylcholine (DPPC) molecules, immersed in water. These phospholipids are organic molecules with a hydrophilic head and a hydrophobic tail, which orient themselves towards each other in water such that they form stable bilayer sheets. These bilayers constitute the outermost part of a cell’s membrane. It is an interesting biophysical question how stable phospholipid bilayers are under shock conditions, because this issue is connected with the question how a cell membrane can be reversibly ruptured to facilitate drug transport to its interior [28].

From a computational perspective, simulating the equilibrium behaviour of phospholipid bilayers is rather efficient. Due to their almost two-dimensional geometry only small volumes of solvent above and below its plane need to be accounted for. However, the simulation of shock phenomena in such systems requires much larger volumes perpendicular to the bilayer plane, as the solvent itself is needed for transporting the shock front and the subsequent release (or rarefaction) wave to the bilayer. Explicit simulation of this solvent requires a much larger part of computational resources than actually spent on the bilayer, even though one is, in general, not interested in the structure of the solvent. This computational bottleneck is so severe that the largest atomistic simulations published to date [29], [30] only considered a system size of 128 phospholipids, with simulation box lengths parallel, and perpendicular to the bilayer plane of 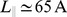 and 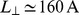, i.e., comparable to the bilayer thickness which is 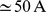.

**Figure 7:**
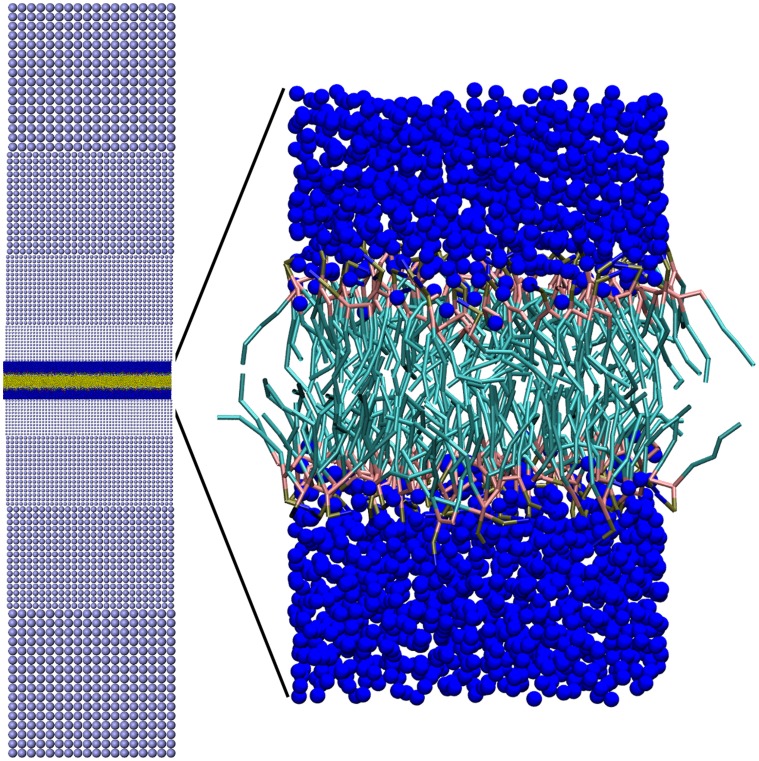
Multi-scale simulation setup. The left part shows the entire simulation domain with dimensions 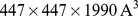. Starting from top and bottom, the continuum domain is represented by SPH particles (light blue) which decrease in mass towards the center of the simulation domain. At the center, a coarse grained MD domain is located. Herein, a phospholipid bilayer (yellow, red, and green) is solvated in water (dark blue). The right part of this figure zooms in on the MD domain, showing blue water and the structure of the bilayer, with hydrophilic lipid head groups (red) and hydrophobic lipid tails (cyan). The simulation domain contains 173264 MD and 150808 SPH particles. Periodic boundary conditions are applied along 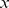 and 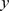, but not along the 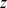-direction.

This situation is ideally suited to a multi-scale approach where we use continuum mechanics to describe the solvent which is far enough away from the lipid bilayer such that there is no direct interaction. Using the tools presented in the preceding section, we employ SPH for this purpose, and couple the atomistic and continuum domains via the DPDE local thermostat. This approach allows us first of all to simulate by far the largest fraction of water using SPH instead of MD. However, as the DPDE thermostat associates an internal energy reservoir and a heat capacity with each MD particle, we are now in the position to employ coarse-grained (CG) MD rather than MD with atomistic resolution to correctly describe a transient process like a shock-wave. This stems from the observation that the temperature increase across a shock-wave front cannot be described using ordinary CG MD, as the heat capacity of such a system would be incorrect, see refs. [31], [32] for further details. In order to further improve computational efficiency, we use a graded resolution within the SPH domain, increasing the smoothing length with increasing distance from the bilayer, see Fig. 7. With this simulation setup, we are able to study shock loading of bilayers with a 64-fold increase in bilayer area compared to the previous studies, using only modest computer resources.

#### Simulation parameters

Within the MD domain, the phospholipid bilayer and surrounding water is described by the MARTINI force field, which maps the atomistic molecular structure of DPPC (chemical formula 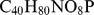), to 12 coarse-grained (CG) beads, connected by bonds, angles, and dihedral forces. Four 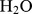 molecules are mapped onto a single CG water bead. Rather than describing this force field here, we refer the read to the original literature [33] and note that it has been used with very good success to describe equilibrium properties and dynamics of phospholipid bilayers [34]. We use the DPDE local thermostat, Eq. (3), on all CG MD particles and set their heat capacity such that the correct atomistic heat capacity is recovered. To give an example, one CG water bead represents four water molecules, with 9 degrees of freedom (DOF) each (rotational and translation DOFs). The CG bead retains 3 DOF due to translation. Therefore, 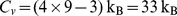. The amplitude of the DPDE forces, 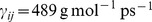, was parametrized by requiring that the temperature rise across a shock-wave travelling with 1000 m/s is the same in both a fully atomistic simulation of SPC water and the CG MD water model used here, a procedure detailed in [32]. All CG MD particles carry a mass of 72 g/mol.

In the continuum domain, we employ Tait’s equation of state,
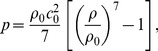
(15)which is capable of describing shock waves with sufficient accuracy for our purposes [35]. Here, 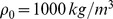 is the reference density and 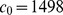 m/s is the sound speed of water corresponding to ambient conditions. SPH is used to discretize the Navier-Stokes equations. We use a non-uniform spatial resolution, with SPH particle masses of {323.9, 982.0, 3065.9, 9239.8} g/mol and corresponding smoothing lengths 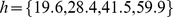 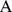. Monaghan’s expression for the artificial viscosity [36] with a coefficient of unity is used to prevent particle penetration. The specific heat capacity of all SPH particles was set to 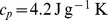, which is accurate for water at ambient conditions.

Mechanical coupling between SPH and MD particles is effected through a combination of both MD and SPH forces, with weighting coefficients of 0.1 and 0.9, respectively. We have determined this combination in a sequence of experiments to give the best results in terms of density and pressure variations at the interface. Thermal coupling is effected via Eq. (10) with a conduction coeffient of 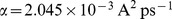, which has been chosen large enough to account for the viscous dissipation in the SPH domain, see Fig. 1. Note that this thermal conductivity coefficient does not represent the macroscopic heat conduction of water. It is an inherent parameter of the coupling algorithm which feeds dissipated energy back into the MD domain.

A Velocity-Verlet time integration scheme is used with a time-step 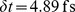. The need for this rather small time-step is caused by the microscopic length scales present within the CG MD domain, and the stability of the DPDE thermostat. For comparison, the Courant-Friedrichs-Lewy condition suggests a time-step 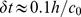, which for the finest SPH resolutions is 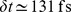. All particles are assigned an internal energy corresponding to a temperature of 310 K, in agreement with a typical warm-blooded physiological temperature.

#### Shock experiments

The setup for our shock-wave experiment is akin to the setup described in dynamic shock propagation test case above: We bound the simulation domain depicted in Fig. 7 from below with a stationary momentum-reflecting mirror. A particle velocity 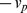 is added to the z-component of all particles. Subsequently, all particles move downwards and pile up against the reflecting mirror. A shock discontinuity at which density, pressure, and temperature suddenly increase moves upwards with speed 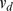. The shock velocity, as measured in a co-moving reference frame, is thus 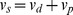. Once the shock reaches the top particles, the system is in the thermodynamic Hugoniot state corresponding to 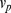. At this point in time, the total linear momentum of all particles is zero. We now remove the momentum reflecting mirror and apply damping boundary conditions to the top and bottom SPH particle layers, with a damping force proportional to particle velocity. This effects a slow release of compression. We choose the damping force to be 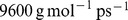. This parameter is quite arbitrary, as in reality it corresponds to the decay of a shock pulse, which in turn depends on the experimental setup. With the chosen value, our computer experiment resembles a laser shock experiment with pulse durations of several picoseconds. Using the damping boundary condition, we simulate the release of the shock-wave for 2500 ps, which is long enough to uncompress and return to the initial density 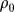.

**Figure 8:**
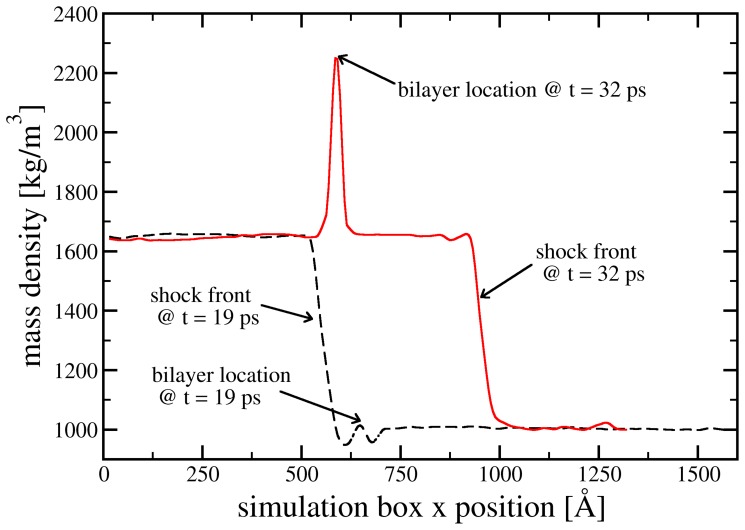
Shock compression of the simulation domain shown in Fig. 7. Starting from an initial mass density 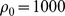 kg/m^3^, a shock discontinuity with speed 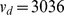 m/s compresses the system into the Hugoniot state with 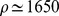 kg/m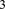. Two different points in time are shown. The peak in the red curve stems from the hydrocarbon tails within the lipid bilayer, which have a lower compression modulus than water.

**Figure 9:**
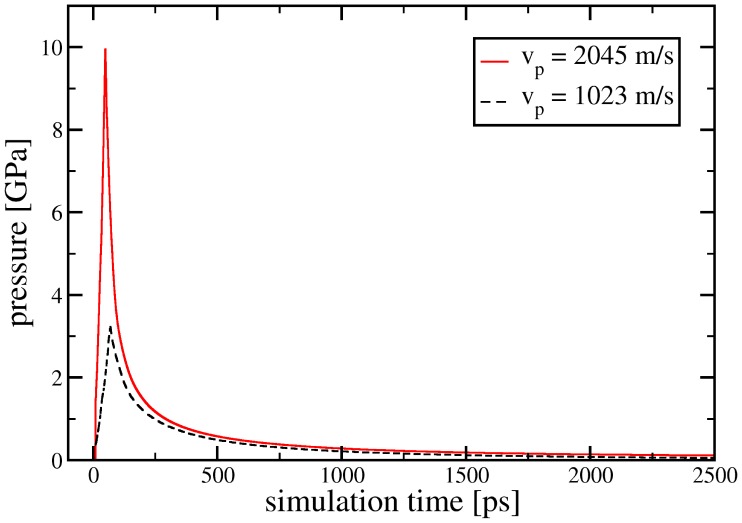
Pressure evolution during shock compression and subsequent release. Data for two piston velocities, 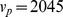 m/s and 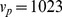 m/s are shown, which correspond to shock-wave velocities of 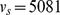 m/s and 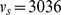 m/s.

We use two different initial particle velocities: 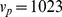 m/s and 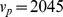 m/s. This results in corresponding shock-wave speeds are 3360 m/s and 5081 m/s, in satisfactory agreement with published data for the Hugoniot slope 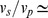 1.8–2.0 [37]. Shock loading leads to a mass density in the shocked state of 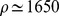 kg/m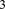 and 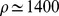 kg/m^3^, for 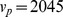 m/s and 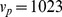 m/s, respectively. The density profile during shock compression is shown in Fig. 8 for 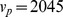 m/s, while the time evolution of the overall pressure in the system is shown in Fig. 9 for both 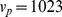 m/s and 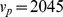 m/s.

We note that the here considered initial particle velocities effect large peak pressures on the order of a few GPa, which decay non-exponentially, on the order of 1000 ps, to less than 10% of their peak values. As a comprehensive, quantitative damage analysis of the lipid bilayer structure is out of scope for this paper, we discuss in the following the induced damage on a qualitative basis using simulation snapshots. In this context we note two features of the equilibrium lipid bilayer structure which is depicted in the right part of Fig. 7: i) no water is present within the region between the two layers of head groups, i.e., where the hydrophobic tails are located. ii) The individual lipids are oriented with their hydrophobic tail such that they point away from the surrounding water, to the inside of the bilayer.

**Figure 10:**
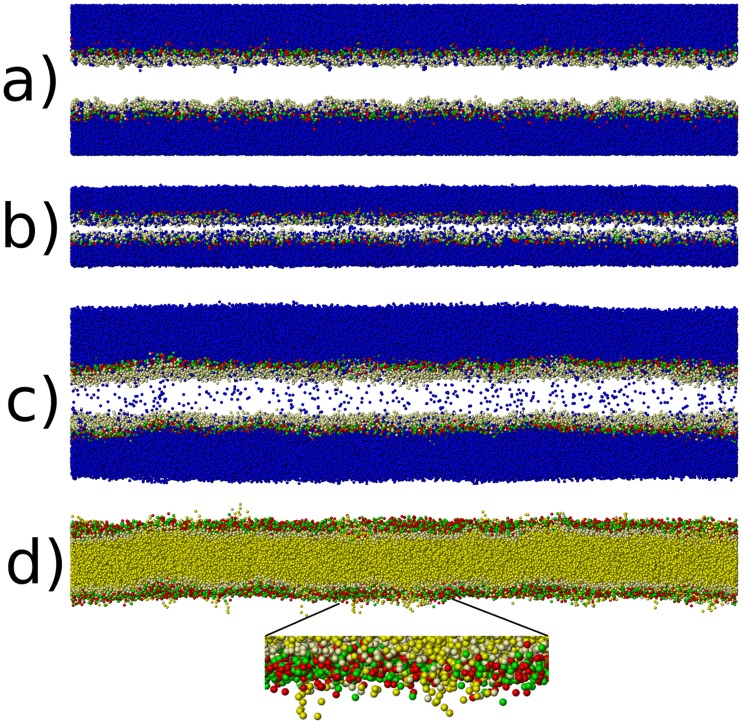
Simulation snapshots for the shock experiment with 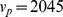 m/s. Structure a) shows the initial equilibrium configuration at 310 K and 1 atm. Only water (blue) and the hydrophilic head groups of the lipids (red, green, and beige) are shown. The hydrophobic tails of the lipids are not shown in order to facilitate the visualization of water within the lipid bilayer. b) shows the state at maximum compression. c) shows the unloaded state after 2500 ps. Note the amount of water introduced into the bilayer. d) also shows the final state after unloading, but here only lipids with their tails (yellow) are shown but no water. Note that some lipid have turned around and now point into the water with their hydrophobic tails, a feature not present in the equilibrium structure (cf. Fig. 7).

**Figure 11:**
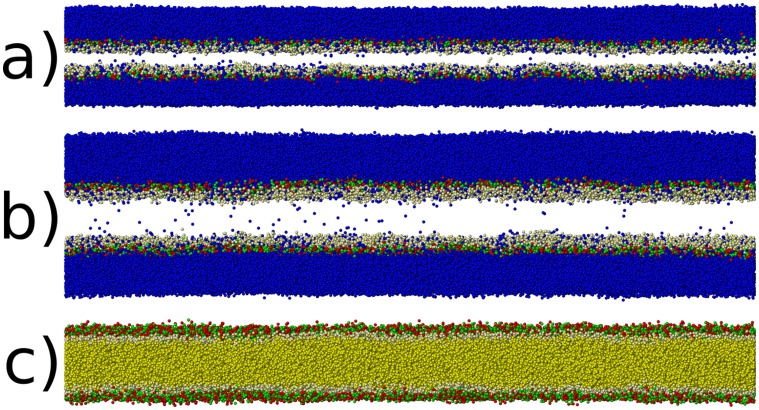
Simulation snapshots for the shock experiment with 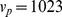 m/s. a) shows the the lipid bilayer at maximum compression. Only water (blue) and the hydrophilic head groups of the lipids (red, green, and beige) are shown. The hydrophobic tails of the lipids are not shown in order to facilitate the visualization of water within the lipid bilayer. b) shows the unloaded state after 2500 ps. Note the smaller amount of water introduced into the bilayer compared to part c) of Fig. 10. c) also shows the final state after unloading, but here only lipids with their tails (yellow) are shown but no water. Note that there are no lipids which have reversed direction due to shock-loading, in contrast with the faster shock experiment.

Comparing to the unloaded, post-shock state of our simulations now, we find for the shock-wave with 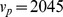 m/s (see Fig. 10), that water is present within the hydrophobic tail region, and that some lipids point towards the surrounding water with their tails. For 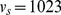 m/s (see Fig. 11), we see less water within the hydrophobic tail region, and do not observe any lipids which have reversed their equilibrium orientation. Our results are in surprisingly good agreement with fully atomistic results [29], which predict water penetration, albeit using a comparatively minuscule simulation size. This study now confirms these findings on a length-scale which is larger by one order of magnitude, thus effectively eliminating a finite-size bias associated with the water penetration mechanism.

## Discussion

In this paper we have presented a method suitable to couple atomistic length-scales, described by MD, with macroscopic length scales described by continuum mechanics, here modelled using SPH. Heat flow between both simulation domains is effected by Fourier heat conduction, and the DPDE local thermostat [17], [18] effects the required correspondence between the continuum definition of temperature (based on internal energy and heat capacity) and particle velocity in the atomistic simulation domain. For transmitting forces between continuum and atomistic domains, we have chosen to employ a simple averaging of MD and SPH forces. Exemplary tests of the here proposed coupling algorithm show that the correct equilibrium probability distribution of particle velocities is obtained in the atomistic simulation domain, indicating that thermal fluctuations have been incorporated in a consistent fashion. The propagation test of a steady planar shock-wave from the continuum simulation domain into the atomistic domain and back demonstrates conservation of energy and shock-wave speed, thus proving that this coupling scheme is stable under shock-wave conditions, i.e., when strong gradients in in pressure and temperature are present.

We have applied our coupling scheme to an interesting biological problem by studying shock loading of a phospholipid bilayer immersed in water. From an application perspective, the important question here is under which shock conditions water penetrates the bilayer such as to weaken it and cause rupture. The bilayer itself and the surrounding water in its immediate vicinity were described using coarse-grained MD, while water further away was modelled using SPH with Tait’s equation of state. This approach constitutes a successful example of a *multi-scale* simulation. It has enabled us to study the water penetration phenomena using a system size which is almost two order of magnitude larger in size and three order of magnitude larger in simulation time than preceding fully atomistic MD simulations [29], [30]. The results of our simulations verify the predictions and observations made in those studies. More importantly, however, our our results remove the uncertainty associated with the finite-size bias of those studies. However, the overall results must be interpreted with some care as we have employed a constant, i.e. temperature-independent, heat capacity. As the coarse-grained Martini model for water and the phospholipids mimics includes intra- as well as intermolecular degrees of freedom, some temperature-dependence of the heat capacity is expected. Additionally, the strong shock conditions could excite quantum-mechanical degrees of freedom which are not accessible at ambient temperatures. Due to the lack of accurate coarse-grained potentials for these temperature- and pressure regimes, we are currently unable to study these effects in detail.

It seems in order to discuss the here proposed coupling scheme in the light of two important contributions to the general context of coupling different length scales and temperature definitions: The SDPD scheme by Español [16] – just like DPDE – incorporates thermal fluctuations and establishes an equilibrium between internal and kinetic temperature. However, in contrast to DPDE, the amplitude of the thermal fluctuations is inversely proportional to the volume associated with a particle, thus allowing to seamlessly bridge between low resolutions, where thermal fluctuations are unimportant, and microscopic resolutions, where thermal fluctuations dominate. While SDPD is very consistent from a theoretical point of view, the approach proposed here is conceptually easier to implement, with clear boundaries between continuum and microscopic domains, and complete freedom of choice as how to integrate the partial differential equations of continuum mechanics, using SPH as we have done, or, e.g. with the method of Finite Elements (FEM).

The FEM-MD coupling strategy devised by Xiao and Belytschko [15] provides a very stringent way of constructing the forces acting between MD and continuum by means of a linear combination of the Hamiltonians of each domain. The linear combination is augmented with a penalty function obtained using the method of Lagrangian multipliers, such that displacements of both MD particles and continuum integration nodes correspond with each other, and that total energy is conserved. Their approach eliminates spurious reflections of pressure waves at the atomistic/continuum interface, which usually present a problem when coupling FEM and MD, but lacks the incorporation of thermal fluctuations. For the here proposed SPH-MD coupling scheme, the mechanical interaction between SPH and MD particles has been chosen as a simple average of the respective Hamiltonians. As the spatial discretization of the SPH continuum domain is identical to the MD particle diameter, spurious oscillations are not pronounced in our scheme, nevertheless we do observe them.

A computational inefficiency of the here presented work is that a single time-step is used for the entire simulation domain. This time-step is determined by the smallest length-scales and largest accelerations, which are found in the MD domain. A CFL criterion for the SPH domain would predict time-step sizes which are order of magnitude larger. It is therefore desirable to employ a multiple-time step algorithm [38]. In combination with coupling SPH to FEM [39], [40], a multi-scale simulation method (both in space and time) for transient phenomena is possible. Future work on our behalf will be devoted to a more sophisticated implementation of the here presented coupling scheme, including a stringent treatment of the mechanical SPH – MD interaction.
